# Probing lasting cryoinjuries to oocyte-embryo transcriptome

**DOI:** 10.1371/journal.pone.0231108

**Published:** 2020-04-06

**Authors:** Binnur Eroglu, Edyta A. Szurek, Peter Schall, Keith E. Latham, Ali Eroglu

**Affiliations:** 1 Department of Neuroscience and Regenerative Medicine, Medical College of Georgia/Augusta University, Augusta, GA, United States of America; 2 Department of Obstetrics, Gynecology and Reproductive Biology, College of Agriculture & Natural Resources/Michigan State University, East Lansing, MI, United States of America; 3 Department of Obstetrics and Gynecology, Medical College of Georgia/Augusta University, Augusta, GA, United States of America; Universite Clermont Auvergne, FRANCE

## Abstract

Clinical applications of oocytes cryopreservation include preservation of future fertility of young cancer patients, substitution of embryo freezing to avoid associated legal and ethical issues, and delaying childbearing years. While the outcome of oocyte cryopreservation has recently been improved, currently used vitrification method still suffer from increased biosafety risk and handling issues while slow freezing techniques yield overall low success. Understanding better the mechanism of cryopreservation-induced injuries may lead to development of more reliable and safe methods for oocyte cryopreservation. Using the mouse model, a microarray study was conducted on oocyte cryopreservation to identify cryoinjuries to transcriptionally active genome. To this end, metaphase II (MII) oocytes were subjected to standard slow freezing, and then analyzed at the four-cell stage after embryonic genome activation. Non-frozen four-cell embryos served as controls. Differentially expressed genes were identified and validated using RT-PCR. Embryos produced from the cryopreserved oocytes displayed 200 upregulated and 105 downregulated genes, associated with the regulation of mitochondrial function, protein ubiquitination and maintenance, cellular response to stress and oxidative states, fatty acid and lipid regulation/metabolism, and cell cycle maintenance. These findings reveal previously unrecognized effects of standard slow oocyte freezing on embryonic gene expression, which can be used to guide improvement of oocyte cryopreservation methods.

## Introduction

Successful oocyte cryopreservation is of interest to preserve future fertility of cancer patients undergoing chemo- and radiotherapy, to substitute embryo freezing toward avoiding associated legal and ethical issues, to prolong childbearing years, and to conserve endangered species. While the discovery of cryoprotective properties of glycerol and dimethylsulfoxide (Me_2_SO) led to successful cryopreservation of many cell types [1, 2], mammalian oocytes have been challenging to cryopreserve due to intracellular ice formation (IIF) [3], cell lysis [4], chemical toxicity of cryoprotective agents (CPA) [5], osmotic stress [6], disruption of cytoskeleton and spindle microtubules [7, 8], premature exocytosis of cortical granules and zona hardening [9, 10], parthenogenetic activation [11–13], and polyploidy [7, 14, 15]. Through intensive efforts to mitigate these cryoinjuries, increasingly encouraging results have been reported with human oocytes after both slow-freezing [16–21] and vitrification [22–25]. A vitrification approach requiring minimum sample volume (less than 1 μl), low permeating CPA concentrations (~30%), and extremely fast cooling/warming rates yielded clinically acceptable results [26–28] and is currently the preferred approach for human oocyte cryopreservation. However, the minimal sample volume, low CPA concentrations, and direct contact with LN_2_ required to achieve extremely fast cooling/warming rates make this approach prone to devitrification, handling and reproducibility issues, and biosafety risk for contaminating cryopreserved samples with different pathogens [29–32]. In contrast, slow-freezing methods are usually not associated with a biosafety risk; however, clinical success rates obtained with slowly frozen human oocytes remain lower than those obtained with the vitrification method [19, 21, 25]. Understanding better the mechanism of cryopreservation-induced injuries may help overcome the shortcomings of the current approaches, and thereby lead to development of more reliable and safer methods for oocyte cryopreservation.

Although lethal cryoinjuries such as IIF and cell lysis are easily recognizable, sublethal injuries may also occur, such as DNA damage, altered gene expression or altered protein function, and may not be obvious immediately. In fact, a significant portion of cryopreserved oocytes usually fail to be successfully fertilized and develop even though they appear morphologically normal after thawing/warming [33]. Understanding sustained effects on embryonic gene expression may help to better understand some sublethal cryoinjuries and associated developmental failure. Past studies investigated the effects of oocyte cryopreservation on whole oocyte transcriptomes [34] [35, 36] or selected genes [37–40] and reported significantly altered mRNA levels. Since the MII oocytes are transcriptionally silent [41, 42] and major embryonic genome activation occurs at the two-cell stage and four- to eight-cell stage in the mouse [43, 44] and human [45], respectively, the published studies do not address how oocytes cryopreservation affect gene expression after embryonic genome activation (EGA) as a way of assessing long-term effects of oocyte cryopreservation.

**Fig 1:**
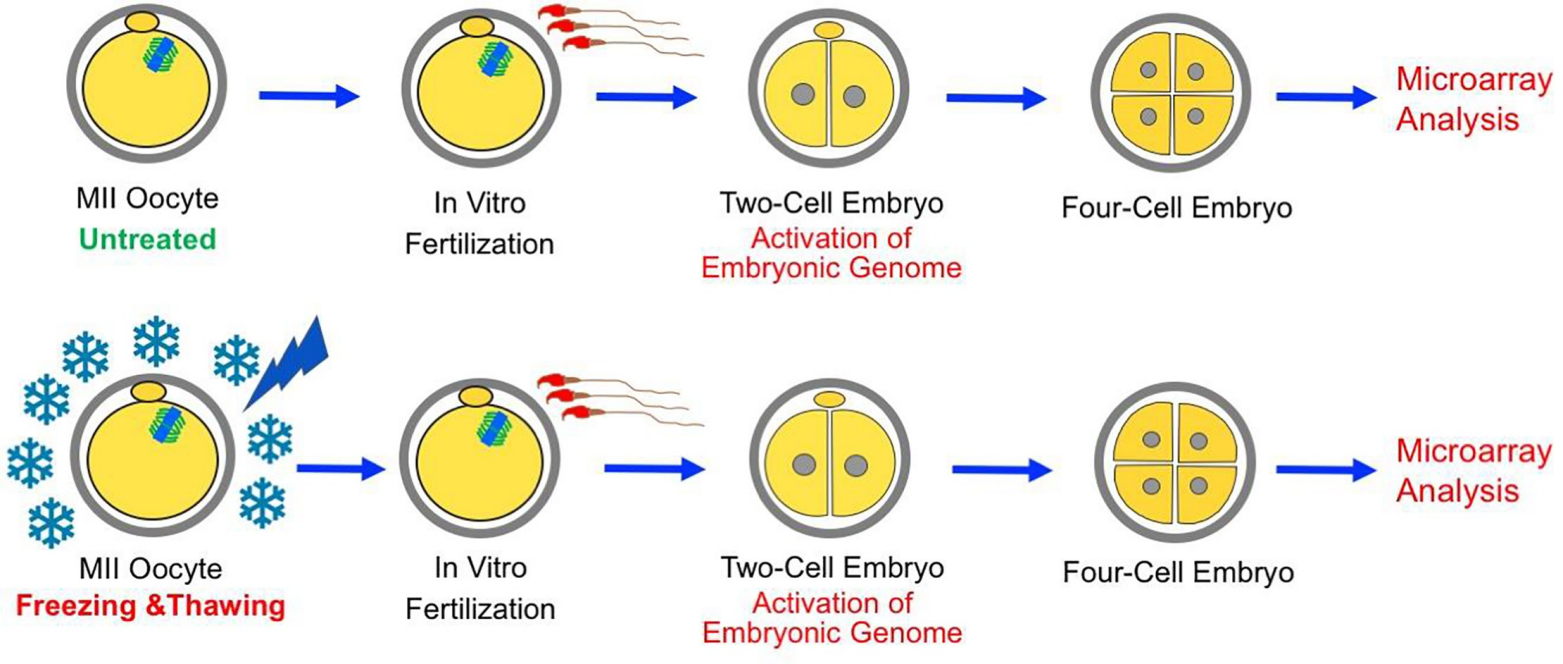
Schematic representation of the experimental design. MII oocytes were frozen-thawed, inseminated, and cultured to the four-cell stage along with untreated controls (lasting cryoinjury). Microarray analysis was performed at the four-cell stage.

The objective of this study was to investigate effects of oocyte cryopreservation on the embryonic transcriptome after EGA. The so-called standard slow freezing method [46–48] is known to induce more significant injuries compared to the vitrification approach. Therefore, we reasoned that the standard slow-freezing protocol would allow us to better detect diverse cryoinjuries including the subtle ones and be the first logical step to establish a basis. Thus, mouse metaphase II (MII) oocytes were frozen and thawed using the standard slow freezing method, inseminated, and compared to control embryos at the four-cell stage using microarrays (Fig 1). Bioinformatics methods such as Ingenuity Pathway Analysis (IPA), Functional Annotation, PPI Network Analysis and Hub Gene Identification were used to reveal up- and downregulated pathways in response to cryopreservation-induced injuries. Results reported here reveal significant effects of oocyte cryopreservation on embryonic gene expression pattern that may impact embryo developmental potential. These effects provide novel biomarkers that may guide improvements of oocyte cryopreservation methods.

## Materials and methods

### Oocyte isolation

All animal care and use protocols were reviewed and approved by the Institutional Animal Care and Use Committee (IACUC) at Augusta University (Protocol # 2009–0032), and the reported experiments were performed according to the IACUC’s guidelines. All animals were maintained under the standard conditions (i.e., 14 h light/10 h dark cycle, at 18–23°C, and 40–60% humidity) with free access to water and food. To harvest a large cohort of metaphase II (MII) oocytes, five to eight-week old B6D2F1 (C57BL/6NCrl X DBA/2NCrl, Charles River Laboratories, Wilmington, MA) females were superovulated by intraperitoneally injecting first a combination of 5 IU equine chorionic gonadotropin (eCG) and 2.5 IU human chorionic gonadotropin (hCG) (PG 600, Intervet, Millsboro, DE) around at 6:00 p.m. and 48–49 hours later, 7.5 IU hCG alone (Sigma, St Louis, MO). Approximately 14 hours after hCG injection, mice were euthanatized using carbon dioxide inhalation followed by cervical dislocation. The oviducts were excised and oocyte-cumulus masses were released from the ampulla into HEPES-buffered Hypermedium [49] under a stereomicroscope. To remove cumulus cells, the oocyte-cumulus masses were treated with 120 IU /ml of bovine testis hyaluronidase (Type IV-S) in phosphate-buffered saline (PBS, Sigma, St Louis, MO) at ambient temperature for 3–4 min and then washed in HEPES-buffered Hypermedium before mechanically removing remaining attached cumulus cells using finely pulled sterile glass capillaries. Afterwards, the cumulus-free oocytes were washed in HEPES-buffered Hypermedium again and then transferred to bicarbonate-buffered Hypermedium for recovery at 37°C before experiments.

### In vitro fertilization and embryo culture

In vitro fertilization (IVF) and culture of fertilized eggs were carried out as described previously [13]. Briefly, the cauda epididymides of a 4- to 6-month old mature BDF1 male (Charles River Laboratories) were aseptically dissected and placed in a large drop (0.4 ml) of preequilibrated BSA-free Hypermedium. Sperm were then released into the medium by gently puncturing the cauda epididymides with a hypodermic needle and dispersed at 37°C for 15 min. Subsequently, to have 1-2x10^6^ sperm/ml, an appropriate volume of the sperm suspension was added to insemination drops containing 70 μl of Hypermedium with BSA supplementation. To capacitate sperm, the insemination drops were incubated at 37°C under a humidified atmosphere of 5% CO2 in air for 1 to 2 hours. Next, oocytes were introduced into the insemination drops and incubated under the same conditions for 5–6 hours to achieve IVF. After insemination, the oocytes were washed and cultured in Hypermedium. Cleavage to the two-cell stage was examined after overnight culture while development to the four-cell stage was evaluated after 40 hours of culture.

### Oocyte cryopreservation

MII oocytes were cryopreserved using the standard slow freezing method [46–48]. Briefly, PBS containing 10% heat inactivated fetal bovine serum (FBS) (HyClone, Pittsburgh, PA) was used for preparation of cryopreservation solutions. MII oocytes were first loaded with dimethylsulfoxide (Me_2_SO) by incubating them in 1.5M Me_2_SO at room temperature (RT) for 10 min, and then transferred to the final cryopreservation solution containing both 1.5M Me_2_SO and 0.1M sucrose for additional 5 min at RT. During this step, oocytes were aspirated into sterile 0.25-cc straws (TS Scientific, Perkasie, PA) that were introduced into a controlled-rate freezer (KRYO 10 Series III, Planer, Middlesex, UK) at the end of the final CPA loading step. Next, the samples were cooled to -7°C at 2°C/min and held at that temperature for 10 min upon manual seeding of extracellular ice. At the end of the holding period, the samples were cooled to -35°C at a rate of -0.3°C/min before being plunged into liquid nitrogen for storage.

For thawing, straws were removed from liquid nitrogen, kept on air for 15 sec and then immersed in a water bath at 37°C until ice disappeared. Next, the content of each straw was released into an empty dish, and Me_2_SO was removed by successively transferring oocytes to its decreasing concentrations (i.e., 1.0M Me_2_SO + 0.1M sucrose; 0.5M Me_2_SO + 0.1M sucrose; and 0.0M Me_2_SO + 0.1M sucrose) at RT with 7-min intervals. Upon removal of Me_2_SO and sucrose, oocytes were rinsed in Hypermedium before being transferred to a fresh drop of Hypermedium for recovery at 37°C. Post-thaw survival of cryopreserved oocytes was assessed after a 60-min recovery period by morphological criteria [13].

### RNA preparation and microarray hybridization

For total RNA isolation, 20 four-cell embryos were lysed for each sample by transferring them in 0.5 μl of PBS containing 0.01% polyvinyl alcohol (PVA, Sigma) to 19.5 μL PicoPure buffer (PicoPure^™^ RNA Isolation Kit, Arcturus, Mountain View, CA). Next, samples were heat treated at 42°C for 30 min and then stored at -80°C until isolation of total RNA from each sample using PicoPure^™^ RNA Isolation Kit according to the manufacturer’s protocol, including a DNAse treatment (RNase-Free DNase Set, Qiagen, Germantown, MD).

For gene expression profiling, Affymetrix Mouse Genome 430 2.0 array (Affymetrix, Santa Clara, CA) that covers 34,000 well-substantiated mouse genes were used. Briefly, total RNA isolated from each sample was amplified using RiboAmp Plus RNA Amplification Kit (Arcturus). The subsequent steps including the second round of amplification and labeling, fragmentation, hybridization, and scanning of the microarray slides were performed by the Genomics Core Facility at Temple University according to the manufacturer’s instructions. RNA purity and concentration were evaluated by spectrophotometry using NanoDrop ND-1000 (ThermoFisher). RNA quality was assessed by the Agilent 2200 TapeStation (Agilent Technologies) and assured of an RNA Integrity Number (RIN) ≥ 7. Total RNA samples were processed using the GeneChip 3’ IVT Reagent Kit (Affymetrix) according to the manufacturer’s protocol. After 16 hours of hybridization, the arrays were washed and stained using Affymetrix GeneChip Fluidics Station 450 systems. The stained arrays were scanned on an Affymetrix GeneChip Scanner 3000. Data were obtained in the form of CEL files. The CEL files were imported into R with the Affy package [50]; normalization and correction were conducted with the GCRMA methodology.

### Real-Time Polymerase Chain Reaction (RT-PCR)

To confirm gene expression profiles obtained from microarray analyses, a quantitative RT-PCR analysis was performed on selected genes. RNA extraction and cDNA preparation from control and treated four-cell mouse embryos (20 embryos per sample) were carried out using the Power SYBR-Green Cells-to-Ct Kit (ThermoFisher Scientific) according to the manufacturer’s protocol. To eliminate DNA contamination, the samples were treated with DNAse supplied by the Cells-to-Ct Kit. cDNA was then used as a template to compare gene expression profiles between the groups (Fig 1). Each group had 3 samples and each sample was analyzed in duplicates. The signals were detected with SsoAdvanced Universal SYBR Green Supermix (BioRad) using the LightCycler 96 detection system (Roche). The PCR program started with an initial denaturation step for 3 minutes at 95°C, followed by 40 cycles of 10 seconds at 95°C, 30 seconds at 60°C and a melting curve analysis from 65°C to 95°C. The data was analyzed with the LightCycler 96 SW 1.1 software and normalized against to the housekeeping gene Peptidylprolyl isomerase A (Ppia) which was amplified in the same run [51]. The primer sequences are given in Table 1.

**Table 1:** Primer pairs used for gene expression analysis by RT-PCR.

Name	Forward Primer (5’-3’)	Reverse Primer (5’-3’)
**Allc**	TCCTCGCATGTCAATCCAAG	TCAGTAACGGCTTCAAACTCC
**Cpa1**	GTCTACACCCACAAAACGAATC	ACGGTAAGTTTCTGAGCAGG
**Gsto**	TTTCCAGATGACCCGTACAAG	GAGTCTTCCTTTCTCTTCGACC
**Ly6A**	GGATGGACACTTCTCACACTAC	GCAGGTAATTGATGGGCAAG
**Oosp1**	AGAGTCCTCATTTCTGTGAAGC	GGTGATCTTCGCTTGATGTTG
**Phlda3**	CCGTGGAGTGCGTAGAGAG	TCTGGATGGCCTGTTGATTCT
**Pign**	TTTGCTTTGGGATTGCTTATCCA	GTATCTGCTCTCAGGCCATCA
**Ppia**	CGCGTCTCCTTCGAGCTGTTTG	TGTAAAGTCACCACCCTGGCACAT

#### Statistics

Experiments were repeated at least three times, and data presented are means of experimental repeats with error bars representing standard-error of mean (SEM). GraphPad Prism (GraphPad Software, Inc., San Diego, CA) was used to analyze the viability and cleavage rates by ANOVA and Tukey’s pairwise comparison test. Arcsine transformation was performed on proportional data before ANOVA. Differences between the groups were considered significant at p<0.05. *P-*values or the level of significance are stated in the figure legends. Differential expressions of genes were calculated with the limma package [52]. The Benjamini & Hochberg adjusted p-value was utilized, significance was set at FDR < 0.05.

#### Functional analysis

Differentially expressed genes were analyzed by several approaches to assess the possible impact of altered patterns of embryonic gene expression on embryo biology. WebGestalt [53] was utilized for an overrepresentation enrichment analyses for the GO categories of biological processes, molecular functions, and cellular components, and pathways via Kyoto Encyclopedia of Genes and Genomes (KEGG) pathway analysis and Reactome. The p-value (p<0.05) was set as the cut-off. Combined lists of up- and down-modulated genes were submitted to Qiagen Ingenuity Pathway Analysis® for analysis of affected canonical pathways (CPs), diseases and functions (DFs), and upstream regulators (URs). For CP and DF analyses, IPA takes into account the number of DEGs and the number of molecules in the knowledge database associated with that CP or DF category, and the total number of DEGs and the number of molecules in the knowledge database. For UR analysis, IPA takes into account the number of DEGs regulated by a given UR. The analyses reveal significant associations of DEGs with CPs, DFs, or URs (p-values) as well as predicted directionality, if any, reflected in z-scores. The z-score reflects activation (z > 0) or inhibition (z < 0), with z > 1.96 indicating significant activation or increase, and z < −1.96 indicating significant inhibition or decrease. URs can be reported as “affected”, “activated”, or “inhibited” even if not expressed or not altered in expression level.

#### Construction of Protein-Protein Interaction (PPI) networks

PPI networks for the identified DEGs were constructed and visualized using Cytoscape version 3.7.1 [54]. STRING (Search Tool for the Retrieval of Interacting Genes/Proteins) was used to identify interacting proteins with >0.4 confidence score [55]. Network analyzer, a plug-in of Cytoscape, was used to calculate the parameters [56]. Common hub genes were identified using Cytoscape’s CytoHubba application [57]. Top 10 genes were evaluated using 3 local based methods (MNC, MCC, and Degree) and 3 global based methods (EcCentricity, Stress, and EPC) using CytoHubba.

#### The module analysis

The module analysis was performed using Molecular Complex Detection (MCODE), a plug-in of Cytoscape, to identify the interconnected submodules in the PPI network for the identified DEGs with degree cutoff = 2, node score cutoff = 0.2, k-score = 2, and max depth = 100 [58]. Only significant modules with MCODE score ≥4 and node ≥6 were searched. As an alternative approach, Markov Cluster algorithm (MCL), a Cytoscape plugin by clusterMaker [59], was used to predict protein modules with the following parameters: Inflation coefficient = 2.5, weak edge weight pruning threshold = 1E-15, number of iterations = 16, and maximum residual value = 0.001.

## Results

### Cryosurvival, fertilization, and embryonic development

**Fig 2:**
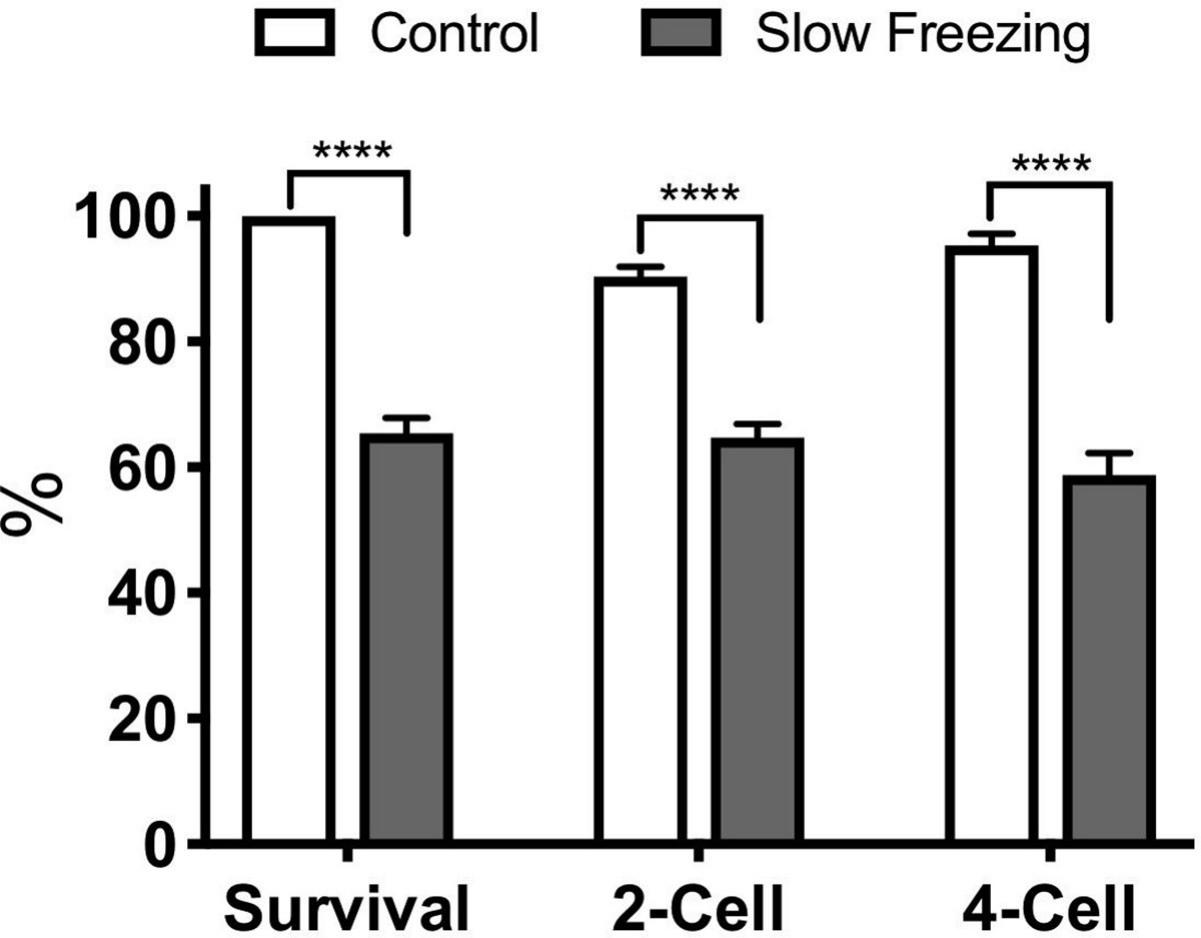
Post-thaw viability, fertilization (2-cell), and early embryonic development (4-cell) of cryopreserved oocytes with respect to untreated controls. Data shown are mean±SEM. ****: P<0.0001.

To study lasting cryoinjuries to active transcriptome, a total of 1,170 MII oocytes were cryopreserved by widely used standard slow freezing. After thawing and a recovery period of one hour at 37°C, 65.4±2.5% of the cryopreserved oocytes (n = 753) remained viable on average (Fig 2). Of 753 frozen-thawed and intact oocytes, 493 were fertilized (mean ± SEM: 65.5±2.1%) as assessed by cleavage to the two-cell stage and 310 of the two-cell embryos developed to the four-cell stage (mean ± SEM: 58.8±3.5%). A total of 311 untreated MII oocytes served as controls. The fertilization and four-cell rates (mean ± SEM) for the controls were 90.3±1.6% (n = 289) and 95.3±1.9% (n = 277), respectively, and significantly higher than those of oocytes underwent cryopreservation (Fig 2).

### Effects of cryopreservation on active transcriptome

All microarray analyses were carried out at the 4-cell stage. The transcriptome data was then used to identify differentially expressed genes (DEGs) as described earlier. Oocytes cryopreserved at the MII stage and analyzed after development to the four-cell stage (lasting cryoinjury to active transcriptome) displayed a total of 335 differentially expressed genes, which mapped to 305 genes in the IPA database (200 up-regulated, and 105 down-regulated) (S1 Table).

#### Functional analysis of the DEGs

**Fig 3:**
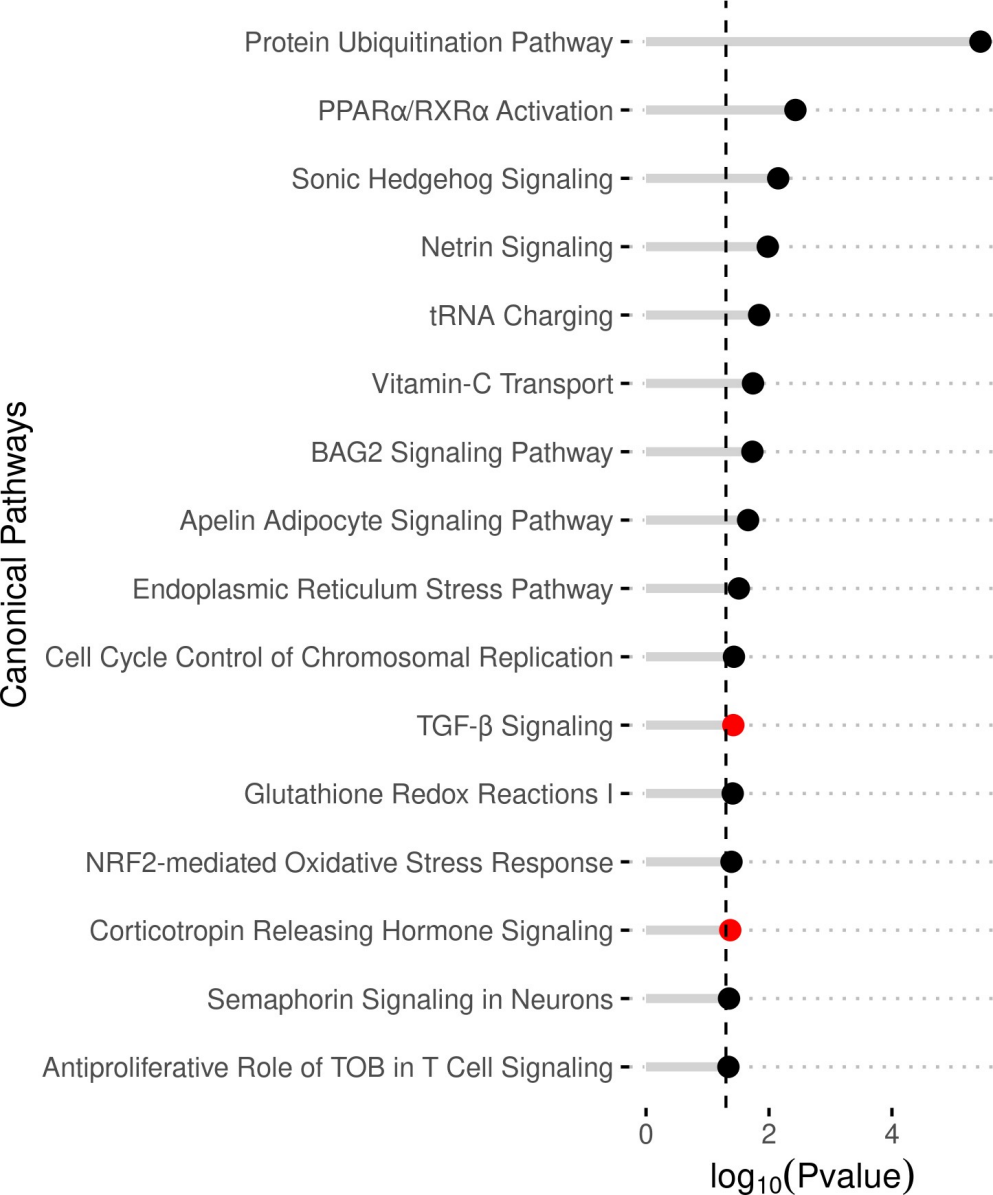
IPA canonical pathway analysis of DEGs. The enriched pathways are given on the y-axis. The x-axis represents the significance (negative Log of P-value). The color of the dots indicated level of significant z-score: black: |z|<1.96, red/activated: z>1.96, blue/inhibited: z<1.96.

**Fig 4:**
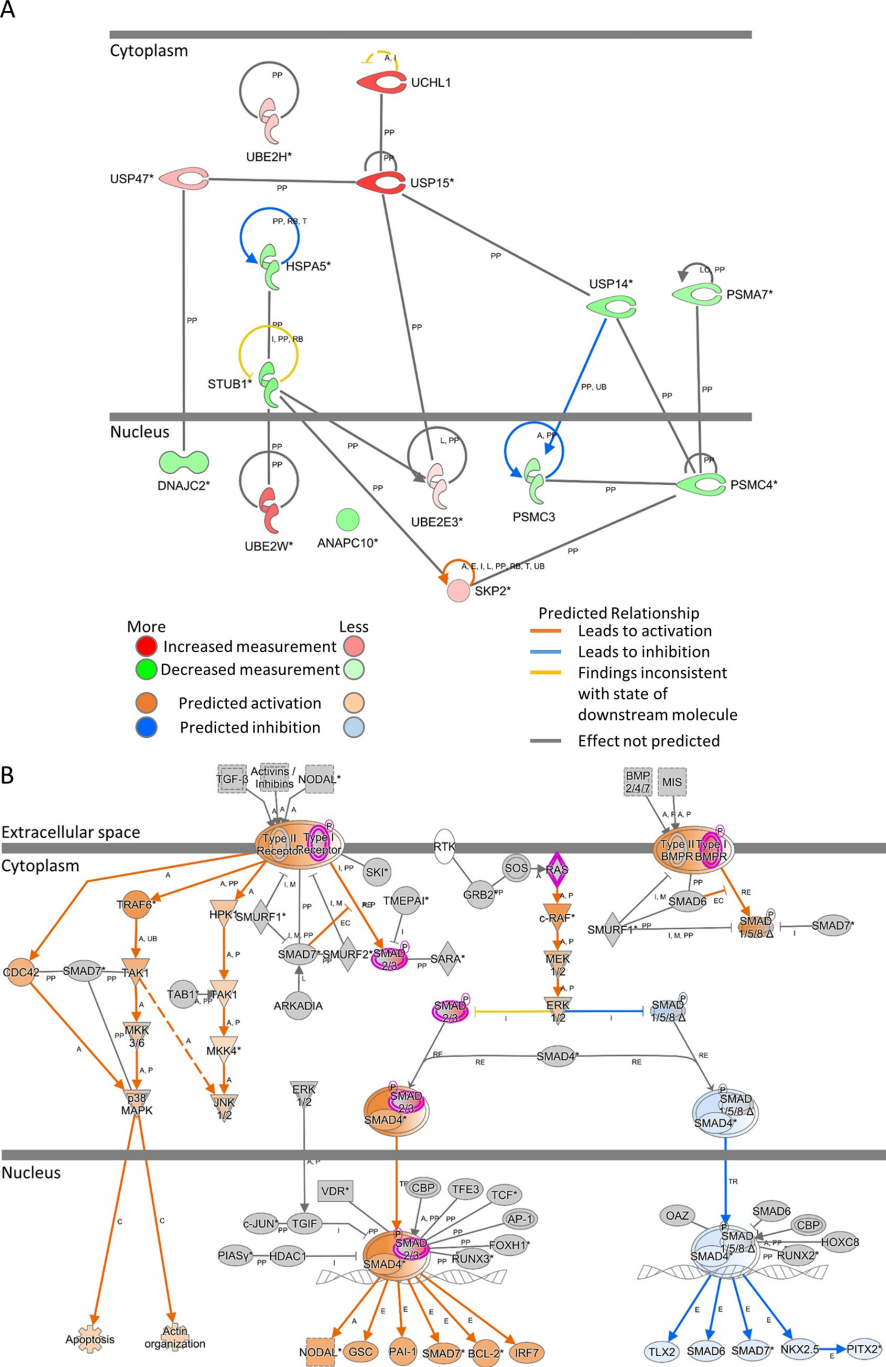
IPA cellular view of: A) 15 affected molecules in the significant Protein Ubiquitination pathway. B) Canonical pathway TGF-β Signaling pathway, coloring indicating measured and predicated states.

*Pathway analysis*. IPA CP analysis yielded 16 significantly affected pathways (S2 Table). Both Corticotropin Releasing Hormone Signaling and TGF-β Signaling pathways were predicted to have significantly activated z-scores, 2.236 and 2.0, respectively. The three CPs with the lowest p-values were: Protein Ubiquitination Pathway, PPARα/RXRα Activation, and Sonic Hedgehog Signaling (Fig 3). To highlight the molecules and their respective positions in the cell, the Protein Ubiquitination and TGF-β Signaling pathways were visualized in Fig 4.

The KEGG-pathway analysis revealed that DEGs in the cryopreservation group were mainly associated with eight significant pathways including: Proteasome, Aminoacyl-tRNA biosynthesis, 2-Oxocarboxylic acid metabolism, Biosynthesis of amino acids, and Citrate cycle (TCA cycle) (S3 Table).

In contrast the Reactome analysis resulted in a much large number of significant pathways. Some of the top returned entries include, Cellular responses to stress, Cellular responses to external stimuli, Regulation of RUNX2 expression and activity, Autodegradation of Cdh1 by Cdh1:APC/C, and Regulation of PTEN stability and activity (S4 Table).

#### Upstream regulator analysis

**Fig 5:**
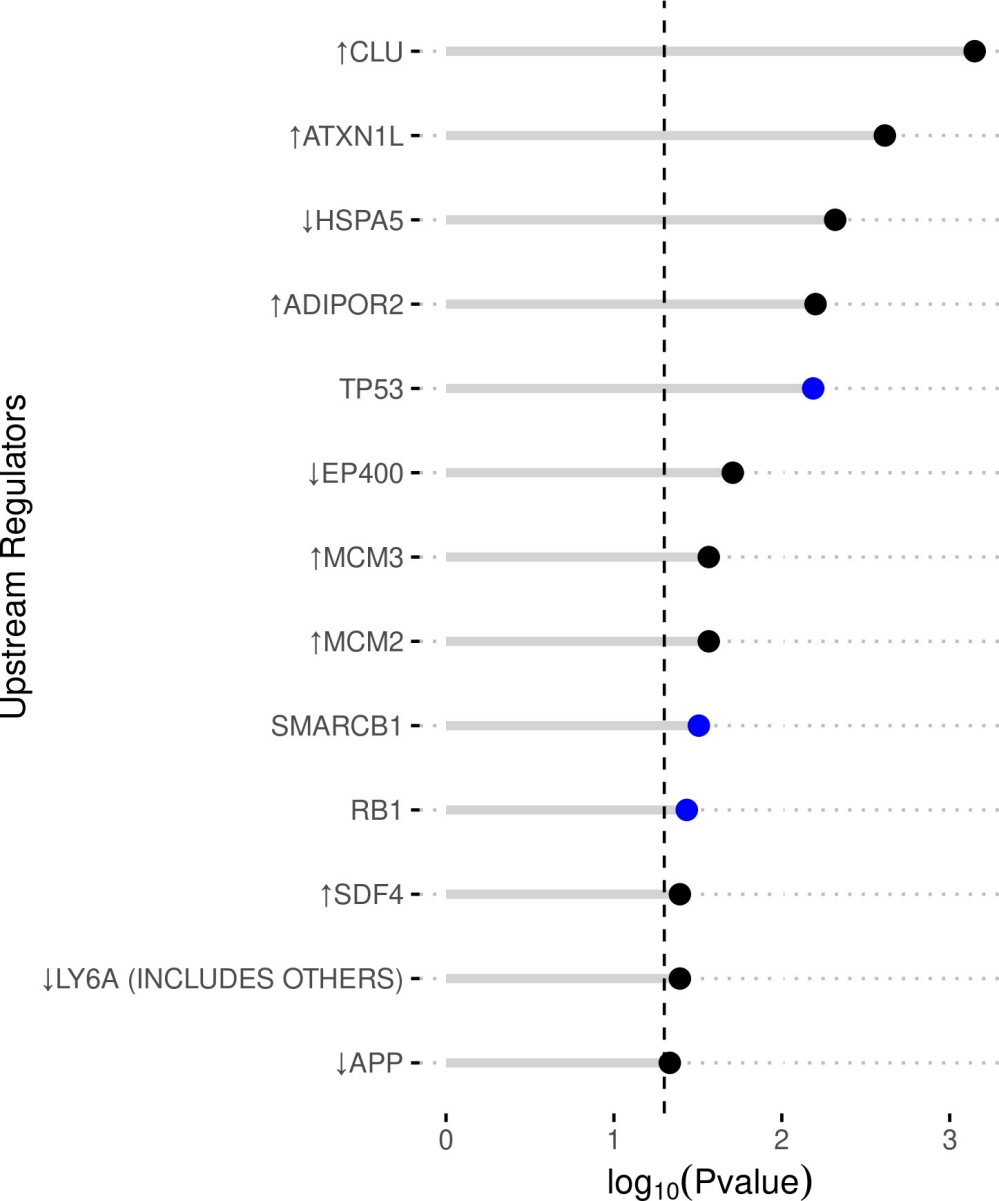
IPA upstream regulator analysis of DEGs. The enriched regulators are given on the y-axis. The x-axis represents the significance (negative Log of P-value). The color of the dots indicated level of significant z-score: black: |z|<1.96, red/activated: z>1.96, blue/inhibited: z<1.96. Arrows preceding the regulator name indicate the direction of differential expression from the cryopreservation group.

The IPA upstream regulator analysis identifies regulators and when applicable, predicts their activity based on their down-stream effectors. Our IPA UR analysis identified 122 affected URs. Three of these URs were predicted to significantly inhibited activity: RB1, SMARCB1, and TP53. Ten significantly affected URs were themselves differentially expressed. Six of these URs had two or more affected downstream molecules (ADIPOR2, ATXN1L, and CLU were upregulated; APP, EP400, and HSPA5 were downregulated) (Fig 5 and S5 Table).

#### Disease and function analysis

**Fig 6:**
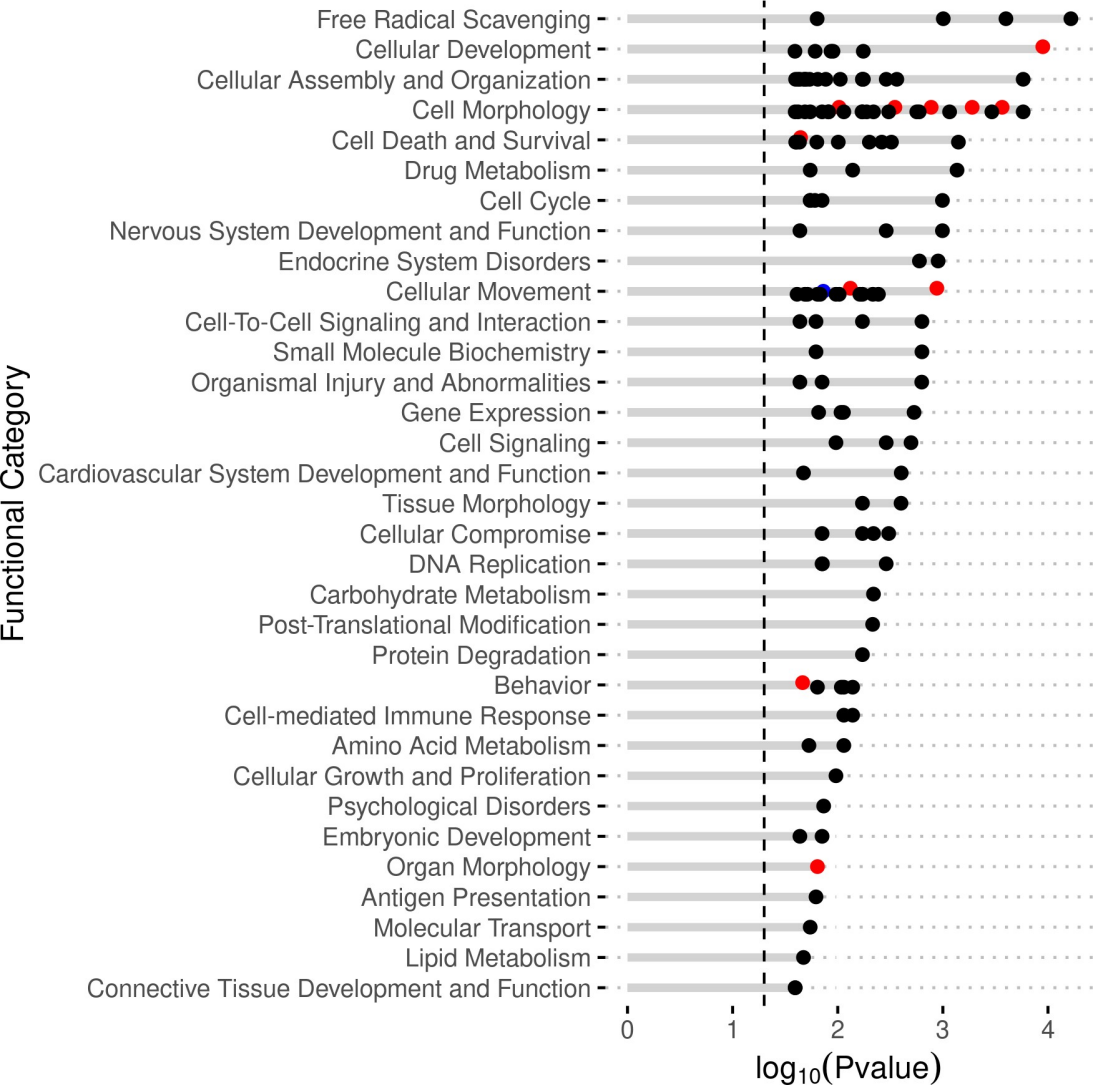
IPA disease and function analysis of DEGs. The enriched functions are given on the y-axis. The x-axis represents the significance (negative Log of P-value). The color of the dots indicated level of significant z-score: black: |z|<1.96, red/activated: z>1.96, blue/inhibited: z<1.96.

There were a number of significant biological functions identified from the IPA analysis (S6 Table). Of note, the biological function Migration of connective tissue cells had a significantly inhibited z-score (z = -2.395). Both Cellular infiltration by macrophages and Cell viability of epithelial cell lines were significantly activated with z-scores of 2.578 and 2.213, respectively. Additional significant functions included with greater than 20 DEGs: Cell death of tumor cell lines, Viral Infection, Expression of RNA, Transcription of RNA, and Transactivation (Fig 6).

#### Gene ontology analysis

GO biological process (GOBP) analysis of the cryoinjury group revealed 19 enriched categories including: mitochondrial gene expression, proteasomal protein catabolic process, cellular amino acid metabolic process, and positive regulation of proteolysis (S7 Table). In terms of cellular component (GOCC), the DEGs were significantly enriched in 6 categories, mitochondrial matrix, peptidase complex, mitochondrial protein complex, chaperone complex, ATPase complex, and organelle inner membrane (S8 Table). For molecular function (GOMF), the DEGs in the cryopreservation group were mainly associated 13 significant entries, the top of which were: coenzyme binding, oxidoreductase activity, acting on the CH-CH group of donors, ribonucleoprotein complex binding, and sulfur compound binding (S9 Table).

#### Construction of PPI network and identification of hub genes

**Fig 7:**
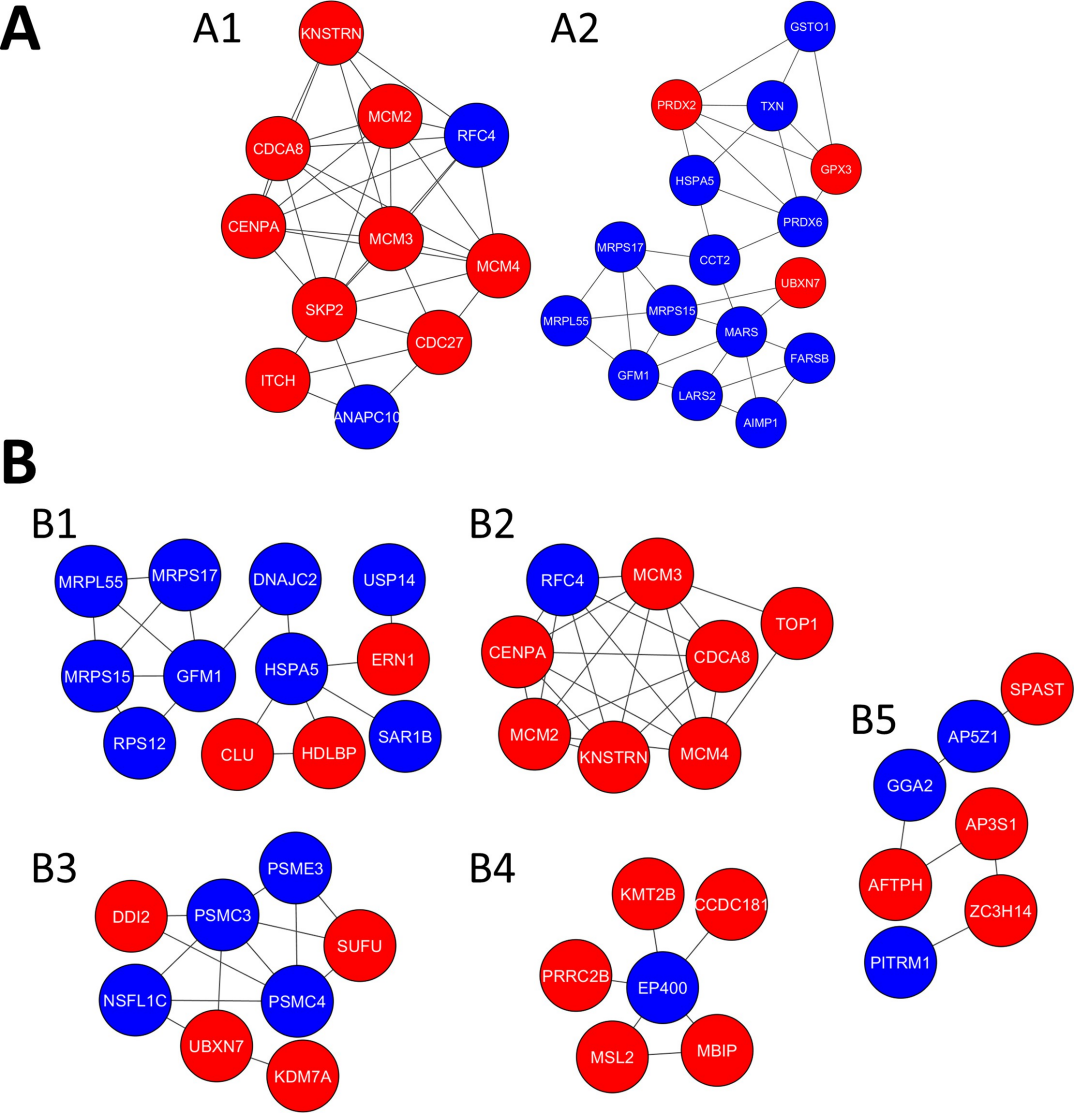
The top clusters obtained from Protein-Protein Interaction (PPI) network analysis. A) MCODE clusters with scores above 4 and had more than 6 nodes. B) The top five MCL clusters, each more than 6 nodes. The interaction networks were visualized by the Cytoscape. Red and blue circles indicate up- and downregulated genes, respectively.

After the upregulated and downregulated DEGs were submitted into STRING online database, the PPI networks were constructed with a confidence score >0.4. The PPI network of the cryopreservation group consisted of 301 nodes and 387 edges. Subsequently, top hub genes were identified from the up- and downregulated PPI network using 6 centralities (MNC, MCC, Degree, EcCentricity, EPC and Stress). The top 10 genes for each centrality are shown in Table 2. The results revealed that a total of 19 hub genes were identified as a result of lasting cryoinjury to active transcriptome. When up- and downregulated DEGs were subjected to the MCORE plug-in of Cytoscape, two modules scored above 4 and had more than 6 nodes (Fig 7A). We next used MCL because it seems to yield more accurate predictions than other algorithms [60]. Of 64 clusters identified, five had more than 6 nodes (Fig 7B).

**Table 2:** The top ten ranked hub genes obtained from CytoHubba analysis based on 6 different centralities.

MCC	MNC	Degree	EC	Stress	EPC
PSMA7	PSMA7	UBXN7	WSB2	UBXN7	PSMA7
UBE2E3	UBE2E3	PSMA7	APP	PSMA7	ITCH
ITCH	ITCH	ITCH	UBE2E3	ITCH	CDC27
CDC27	CDC27	CDC27	ITCH	STUB1	ANAPC10
ANAPC10	ANAPC10	ANAPC10	CDC27	CCT2	STUB1
STUB1	SKP2	STUB1	STUB1	SKP2	SKP2
SKP2	PSMC3	PSMC3	SKP2	PSMC4	PSMC3
PSMC3	PSMC4	SKP2	PSME3	MARS	PSMC4
PSMC4	PSME3	PSMC4	RSP12	UBE2H	PSME3
PSME3	MCM3	UBE2H	HSPA5	HSPA5	UBE2H

MCC: Maximal Clique Centrality; MNC: Maximum Neighborhood Component; EPC: Edge Percolated Component; EC: EcCentricity; DEG: Differentially expressed genes

#### Validation of microarray data

**Fig 8:**
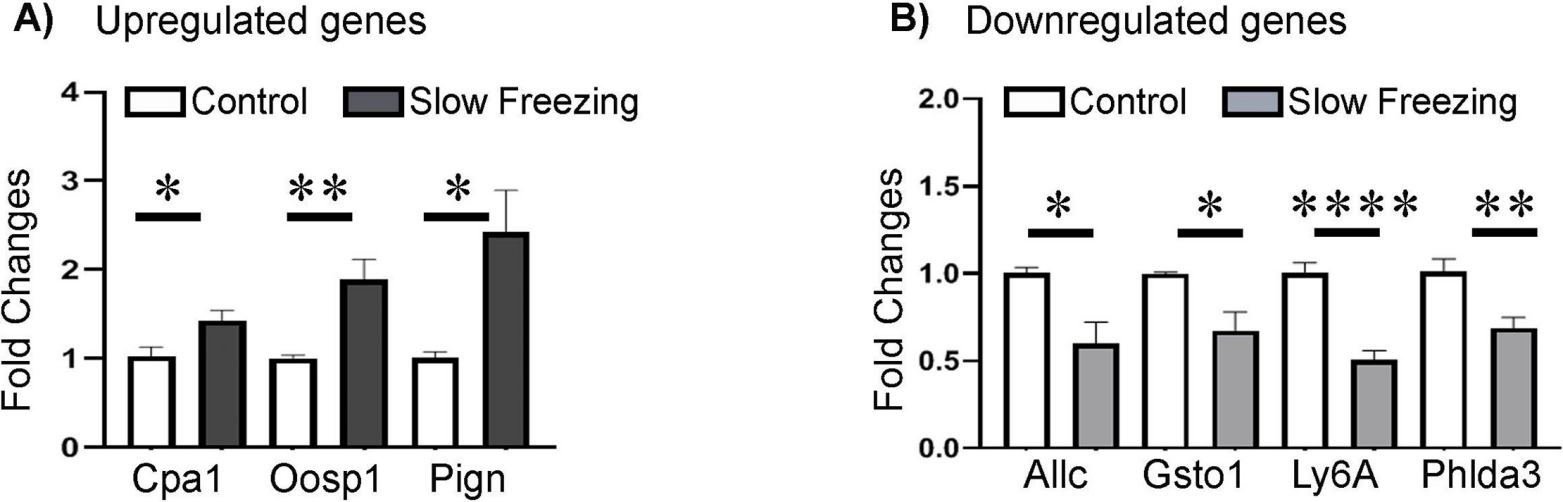
Validation of microarray data by RT-PCR. The mRNA levels of three upregulated **(A)** and four downregulated genes **(B)** determined by microarray analysis were quantified by RT-PCR relative to Ppia expression in the control and cryopreservation groups. * = p<0.05; ** = p<0.01; **** = p<0.0001.

Quantitative RT-PCR was used to verify the microarray results. A total of 7 genes were selected based on their highly differentiated expression in microarray analysis. The expression of these genes was normalized to the expression of the housekeeping gene *PPIA*. When compared to the control group, the expression of *CPA1* (1.43 fold), *OOSP1* (1.89 fold), and *PIGN* (2.43 fold) was significantly increased in the lasting cryoinjury group, whereas the expression of *ALLC* (1.66 fold), *GSTO1* (1.48 fold), *LY6A* (2.4 fold), and *PHLDA3* (1.45 fold) was significantly reduced (Fig 8A and 8B) consistent with the microarray data.

## Discussion

By performing standard freezing and thawing at the MII stage, followed by in vitro fertilization and embryo culture to the four-cell stage, and then by subjecting the resulting 4-cell embryos to microarray analysis, this study reveals that cryopreservation induces lasting changes to the embryonic transcriptome along with predicted effects on embryo physiology and developmental potential. To probe cryoinjuries, the standard slow freezing method was chosen in the present study because it is known to induce significant injuries immediately after thawing and subsequently during fertilization and development. Indeed, the post-thaw survival, fertilization, and cleavage to the four-cell stage were significantly lower in the cryopreservation group with respect to untreated controls (Fig 2), suggesting a significant cryoinjury, and thus suitability of this method to probe cryoinjuries.

To study acute cryoinjuries to the oocyte transcriptome, microarray analysis should ideally be performed short after freezing and thawing of MII oocytes. However, mammalian MII oocytes are known to be transcriptionally silent and to use maternal mRNAs until activation of embryonic genome [41, 42]. Consequently, microarray analysis at the MII stage would primarily show degradation of maternal mRNAs but not reveal the acute effect of cryopreservation on active transcriptome. Considering that the major activation of the transcriptional machinery occurs at the 2-cell stage in the mouse [43], the 4-cell stage was selected in the present study to decipher cryoinjuries to transcriptionally active genome. Hence, the present study reveals previously unexplored cryoinjuries to the active transcriptome.

Previous studies on slowly frozen-thawed and vitrified MII oocytes revealed that both cryopreservation methods lead to the reduction in the mRNA content of some genes involved in chromosomal structure maintenance, DNA repair, cell-cycle regulation, cellular response to stress, and ubiquitination pathway [34–36]. Although there are similarities between the findings of the studies mentioned above and our results, a number of dissimilarities also exist. For instance, the transcript level of certain genes was low (*Creg1*, *Pfkfb2*) as a result of cryopreservation in both previously published studies and our study while an opposite (e.g., *Szt2*, *Cstf2t*, *and Cenpa*) or no effect (e.g., *LY6A*, and *OOSP1*) of cryopreservation has been observed on many transcripts. These dissimilarities are probably due to differences in the timing of the transcriptome analysis. The aforementioned three studies [34–36] assessed the transcript levels at the MII stage shortly after cryopreservation, whereas our study examined the transcriptome at the four-cell stage after activation of embryonic genome. Since the MII oocytes are normally transcriptionally silent, the up- and downregulated genes reported in the previously published three studies could be explained by the following possibilities: (1) increased degradation of many transcripts by cryopreservation-associated processes (downregulated ones); (2) compared fresh controls, slower degradation of a small number of mRNAs in cryopreserved oocytes resulting in their detection at a higher level (upregulated ones); and (3) demethylation of promoters of some genes by cryopreservation leading to their premature transcription (upregulated ones). The several-fold (2 to 9) smaller number of upregulated genes versus downregulated ones in the three aforementioned studies support the explanations above. In contrast, our microarray analyses yielded approximately 2-fold higher numbers of upregulated genes after cryopreservation compared to downregulated ones, suggesting that we were probing the active transcriptional response. Nevertheless, the cryopreservation-induced changes in the maternal transcript content of MII oocytes, as reported in the previous studies, are likely to contribute to the lasting cryoinjuries to the active transcriptome observed in the present study.

As a result of lasting cryoinjuries to active transcriptome, 305 genes displayed altered expression. These genes impact five major functional categories: mitochondrial function, protein ubiquitination and maintenance, cellular response to stress and oxidative states, fatty acid and lipid regulation/metabolism, and cell cycle maintenance. Effects on these processes/functions were identified in multiple analyses including IPA, Reactome and GO/KEGG tools. Additionally, we identified 19 Hub genes within the DEG list from Cytoscape analysis, and 120 significantly affected IPA upstream regulator genes, of which 10 were themselves differentially expressed. The proteins encoded by these genes occupy special locations within regulatory hierarchies and take on heightened importance for understanding the mechanisms underlying phenotypic changes, and as targets for further study. For example, nine of these hub genes impact protein ubiquitination, the largest affected IPA pathway in terms of number of affected component molecules. Four hub genes encode proteins involved in stress response pathways, which are also prominently affected in GO and IPA analysis. Three of the Hub proteins are also affected IPA upstream regulators (MCM3, APP, and HSPA5), with HSPA5 appearing in the affected protein ubiquitination pathway. Three IPA upstream regulators manifested predicted inhibition in activity (RB1, TP53 and SMARCB1), along with predicted activation of TGF-ß signaling. In addition, we note an especially high prominence in our results of effects on mitochondrial functions, cytoskeleton, cell stress, and cell death.

In the present study, microarray data were validated using quantitative RT-PCR. It is worth to note that fold changes varied to some degree between two methods. For example, our RT-PCR results show that expression of *CPA1* was 1.43-fold increased in the cryopreservation group with respect to the control group while our microarray data suggest more than 5-fold increase. Such differences may reflect different methods for normalization. Nevertheless, both methods yielded similar results in terms of significantly up- and down-regulated genes, confirming that the false discovery rate (FDR) was minimal.

It is useful to consider the effects of oocyte cryopreservation and cryoinjury in the context of embryonic genome activation (EGA) and subsequent modulation of gene expression thereafter. The oocyte genome undergoes extensive remodeling in preparation for meiosis, and as a part of transcriptional silencing and modulating the supply of nuclear encoded mitochondrial proteins and mitochondrial activity [61]. The epigenetic state of the oocyte may then predispose or restrict subsequent embryonic genome regulation as well as impacting metabolism and other essential functions. A series of genome activation events occur in the mouse embryo. The first wave (EGA1) occurs soon after the first cleavage, driven in large part by oocyte-derived factors such as DPPA2 and DPPA4 activating expression of DUX, and subsequently ZSCAN4 and other EGA1 genes [62]. EGA1 is terminated within a matter of just hours, a process that is essential for viability [63]. EGA1 is terminated through the action of SMCHD1 [64] and an E3 ligase that destabilizes maternal factors [62, 65], The next transcriptional wave, EGA2, is the major activation event, with thousands of genes being induced [62, 64]. Subsequently, EGA3 occurs at the 8-cell stage, so that the 4-cell stage (between EGA2 and EGA3), targeted here for analysis, corresponds to a period of relatively little change in gene expression [66, 67], thus providing a stable readout of the fidelity of EGA1 and EGA2. Many essential events underlie this orderly sequence, including mitosis, cell division, metabolic regulation, and correct regulation of cellular membrane dynamics and integrity. The first S-phase establishes the ability for gene transcription to occur, and the second S-phase allows a transcriptionally repressive state to be established; this constitutes the emergence of the fundamental ability of the embryo to regulate gene transcription [62, 68]. Because early actions of oocyte-expressed transcription factors like SMCHD1 impact gene expression and developmental events during later cleavage stages [69] cryoinjuries to the oocyte can exert long-term effects on a variety of processes. The disruptions in the four-cell stage transcriptome profile observed with cryoinjury impact a number of essential processes that are regulated by the newly activated genome, as evidenced by effects on mitochondrial function, cellular dynamics and blebbing, cell division, protein metabolism, and diverse signaling pathways.

In conclusion, oocyte cryopreservation induces lasting injuries to oocytes that affect embryonic gene expression pattern, characterized by distinctly upregulated and downregulated pathways that may explain poor development of frozen-thawed oocytes. This is of significance because only ~2% of cryopreserved human oocytes can develop to term while majority of them survive cryopreservation [70]. Addressing these cryoinjuries may lead to improved oocyte cryopreservation.

## Supporting information

S1 Table All differentially expressed genes in the cryopreservation group compared to control. (XLSX)

S2 Table IPA canonical pathway analysis results. (XLSX)

S3 Table KEGG pathway analysis. (XLSX)

S4 Table REACTOME pathway analysis. (XLSX)

S5 Table IPA upstream regulator analysis results. (XLSX)

S6 Table IPA disease and function analysis results. (XLSX)

S7 Table GO biologic process analysis. (XLSX)

S8 Table GO cellular component analysis. (XLSX)

S9 Table GO molecular function analysis. (XLSX)
